# Case Report: Sellar collision tumor consisting of pituitary adenoma and craniopharyngioma, with literature review

**DOI:** 10.3389/fonc.2026.1742957

**Published:** 2026-07-06

**Authors:** Junhao Jin, Bin Gu, Guojian Wu, Jun Kong, Huilin Cheng, Xiaoying Wei

**Affiliations:** 1 Department of Neurosurgery, Zhongda Hospital, Southeast University, Nanjing, Jiangsu, China; 2 Department of Neurosurgery, Taikang Xianlin Drum Tower Hospital, Affiliated Hospital of Medical School, Nanjing University, Nanjing, Jiangsu, China; 3 Department of Pathology, Zhongda Hospital, Southeast University, Nanjing, Jiangsu, China

**Keywords:** collision tumor, craniopharyngioma (CP), neuroendoscopic transsphenoidal resection, pituitary adenoma (PA), sellar region tumor

## Abstract

Collision tumors harbor two distinct histological features at the same site, and sellar collision tumors of pituitary adenoma (PA) and craniopharyngioma (CP) are extremely rare. This report presents a 64-year-old female with fatigue, anorexia, palpitations, dizziness, and exertional dyspnea. Examinations confirmed pan-hypopituitarism and life-threatening electrolyte disturbances. Preoperative MRI showed a homogeneous lesion indistinguishable from a standard macroadenoma. She underwent neuroendoscopic transsphenoidal resection. Intraoperatively, the tumor exhibited a fibrous, tough consistency with no discernible boundary between components, resulting in a “blind” resection of the collision pathology. Significant cavernous sinus hemorrhage (800 mL) occurred due to severe adhesion. Postoperatively, hormone replacement therapy was administered, with stable recovery and discharge on day 18. Pathology confirmed a collision of Null-cell adenoma and Adamantinomatous CP. This case highlights the diagnostic pitfall of “occult” collision tumors where imaging and surgical texture fail to differentiate components, necessitating comprehensive immunohistochemistry. Few similar cases have been reported, mostly involving non-functional PA and adamantinomatous CP with diverse pathogenesis hypotheses. This case supplements critical clinical data on diagnosis, surgical management, and prognosis of this rare sellar tumor, highlighting therapeutic considerations for neurosurgery clinicians.

## 1 Introduction

Collision tumors are defined as two neoplasms with distinct histological features arising at the same anatomical site. These uncommon lesions have been identified in a diverse range of organs, such as the adrenal gland, pancreas, breast, colon, esophagus, stomach, and uterus (1–10). Notably, the occurrence of collision tumors in the pituitary gland is exceedingly rare, with only a limited number of cases documented in the literature (11–21). Within the sellar region, pituitary adenoma (PA) represents the most prevalent neoplasm, constituting roughly 84.6% of all sellar masses and 10–15% of intracranial tumors. It is followed in incidence by craniopharyngioma (CP, 3.2%), Rathke’s cleft cyst (RCC, 2%), meningioma (0.94%), metastatic tumors (0.6%), and chordomas (0.5%) (22–24). Although CP and PA are both relatively common tumor types among parasellar lesions, the coexistence of these two neoplasms is extremely rare.

In this article, we present a rare case of a sellar collision tumor and use it as an illustrative framework to explore the pathogenetic mechanisms of this entity through a focused review of the existing literature. The aim of this study is to better characterize the biological features of sellar collision tumors and to provide mechanistic insights into their pathogenesis.

## 2 Case presentation

A 64-year-old elderly female patient was admitted to the hospital due to “fatigue and anorexia for 5 days”. During the disease course, she was accompanied by symptoms of palpitations, dizziness, dyspnea on exertion and diarrhea, with a significant weight loss in the past half month.

After admission, relevant examinations were completed, which indicated that the patient had severe electrolyte disturbances such as hyperkalemia and hyponatremia, combined with decreased adrenal cortical function and thyroid function. The specific hormone levels were as follows: serum cortisol 2.1 μg/dL, serum free triiodothyronine (FT3) 1.4 pmol/L, serum free thyroxine (FT4) 5.8 pmol/L, serum thyroid-stimulating hormone (TSH) 0.3 mIU/L; serum prolactin (PRL) 18 ng/mL, serum growth hormone (GH) 0.4 ng/mL, serum follicle-stimulating hormone (FSH) 8.2 mIU/L, and serum luteinizing hormone (LH) 2.5 mIU/L. Based on these findings, hypopituitarism was considered. Notably, the patient presented with severe secondary adrenal crisis, characterized by critical hyponatremia (119 mmol/L) and hyperkalemia (7.4 mmol/L), necessitating urgent stabilization with high-dose hydrocortisone before surgery. After stabilization, preoperative hormone replacement therapy was initiated, consisting of oral hydrocortisone (20 mg every morning and 10 mg every afternoon) and levothyroxine (37.5 μg daily) for endocrine maintenance.

Subsequently, the patient’s condition progressed, and she developed clinical symptoms such as paroxysmal headache and visual impairment (accompanied by bitemporal hemianopia). To confirm the feasibility of surgery, relevant preoperative examinations were completed. Preoperative magnetic resonance imaging (MRI), including contrast-enhanced sequences (**Figures 1A–F**), revealed an enlarged sella turcica with a large, well-demarcated lesion located in the sellar region, extending into the suprasellar area and exhibiting a characteristic waist sign. The lesion showed marked and homogeneous enhancement on contrast-enhanced images. The optic chiasm was compressed, thinned, and elevated. Mild compression and displacement of the adjacent cerebral white matter were observed, along with compression of the suprasellar cistern and the third ventricle. The normal pituitary gland was not clearly identifiable, and the cavernous sinus was mildly compressed. Preoperative computed tomography (CT) scans (**Figures 1G–I**) demonstrated a well-defined mass in the sellar region with heterogeneous density, further delineating the lesion extent and its relationship with surrounding structures. Based on these imaging findings, the tumor was classified as Hardy Grade B, Knosp Grade 2. Ophthalmological specialist examination confirmed that the patient had bitemporal hemianopia.

**Figure 1:**
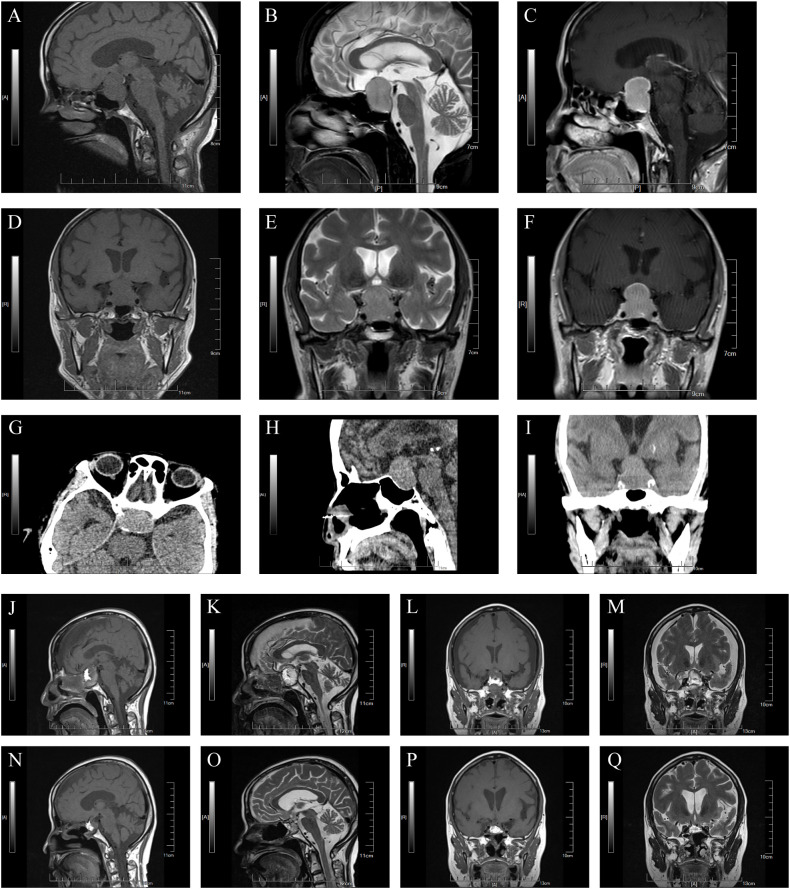
Preoperative and postoperative imaging findings. **(A)** Preoperative sagittal T1-weighted MRI showing the basic morphology of the lesion. **(B)** Preoperative sagittal T2-weighted MRI demonstrating the signal characteristics and extent of the lesion. **(C)** Preoperative sagittal contrast-enhanced MRI showing enhancement of the lesion. **(D)** Preoperative coronal T1-weighted MRI demonstrating the coronal distribution of the lesion. **(E)** Preoperative coronal T2-weighted MRI showing the relationship between the lesion and adjacent brain tissue. **(F)** Preoperative coronal contrast-enhanced MRI further demonstrating the enhancement pattern and extent of the lesion. **(G–I)** Preoperative axial CT images showing the density characteristics of the lesion and its relationship with surrounding structures. **(J)** Postoperative sagittal MRI obtained 15 days after surgery showing postoperative changes at the surgical site. **(K)** Postoperative sagittal MRI obtained 15 days after surgery further demonstrating the postoperative appearance. **(L)** Postoperative coronal MRI obtained 15 days after surgery showing the surgical area and surrounding brain tissue. **(M)** Postoperative coronal MRI obtained 15 days after surgery demonstrating postoperative changes. **(N)** Follow-up sagittal MRI obtained 2 months after surgery showing the postoperative status. **(O)** Follow-up sagittal MRI obtained 2 months after surgery demonstrating a stable postoperative appearance. **(P)** Follow-up coronal MRI obtained 2 months after surgery showing the follow-up findings of the surgical site. **(Q)** Follow-up coronal MRI obtained 2 months after surgery further demonstrating the postoperative condition.

The rechecked hormone levels before surgery showed no significant improvement compared with those at admission, with the specific values being: serum cortisol 2.3 μg/dL, FT3 1.5 pmol/L, FT4 6.1 pmol/L, and TSH 0.28 mIU/L. Considering the progression of the patient’s sellar space-occupying lesion, after excluding surgical contraindications and obtaining informed consent from the patient and her family, a “neuroendoscopic transsphenoidal approach for resection of pituitary lesions” was performed under general anesthesia. During the operation, the tumor presented as a unified, white, and extremely tough fibrous mass measuring approximately 2.5 cm × 2.5 cm × 2 cm. Notably, the interface between the two tumor components was macroscopically indiscernible. Due to the hard consistency, standard suction was ineffective, and sharp piecemeal resection was required. The tumor exhibited tight adhesion to the surrounding tissues, particularly the left cavernous sinus. Dissection in this region precipitated a massive hemorrhage of approximately 800 mL. Hemostasis was successfully achieved by immediately elevating the head of the bed and packing the cavernous sinus with fluid gelatin and gelatin sponge. Following gross total resection, critical structures including the pituitary stalk, optic chiasm, basilar artery, and posterior communicating artery were confirmed to be intact (**Figure 2**). ly, a multilayered skull base reconstruction using a vascularized nasoseptal flap was performed. After the operation, the patient was transferred to the intensive care unit (ICU). On the first day after surgery in the ICU, hormone reexamination showed: after hydrocortisone replacement therapy with oral hydrocortisone (20 mg daily), serum cortisol was 8.5 μg/dL; after starting levothyroxine replacement therapy (37.5 μg daily), FT3 was 2.8 pmol/L, FT4 was 9.5 pmol/L, and TSH was 0.32 mIU/L. After advanced life support treatment in the ICU, the patient’s condition was stable, and she was transferred to the general ward on the 3rd day after surgery.

**Figure 2:**
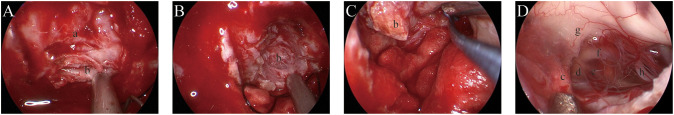
Intraoperative Images **(A)** Tumor exposure **(B)** Intratumoral decompression **(C)** Lateral dissection of the tumor from the cavernous sinus **(D)** Complete tumor resection showing the subarachnoid space **(a)** Sellar floor dura mater; **(b)** tumor; **(c)** pituitary stalk; **(d)** basilar artery; **(e)** superior cerebellar artery; **(f)** posterior cerebral artery; **(g)** hypothalamus; **(h)** anterior communicating artery.

On the 15th day after surgery, imaging reexamination (**Figures 1J–M**) showed postoperative changes of the pituitary tumor, with abnormal signal filling in the sphenoid sinus and sellar region; contrast-enhanced scan showed linear enhancement at the posterior edge of the surgical area; the pituitary stalk and pituitary structure were not clearly visible; patchy effusion signals were seen under the bilateral frontotemporal dura mater. Hormone levels tested on the same day were: under the maintenance dose of hydrocortisone treatment, serum cortisol was 12.3 μg/dL; after adjusting the levothyroxine dose according to previous results, FT3 was 3.0 pmol/L, FT4 was 11.8 pmol/L, TSH was 0.4 mIU/L; serum PRL, GH, FSH, and LH were similar to those on the first day after surgery, with no obvious abnormalities.

Routine pathological examination (**Figure 3A**) suggested “PA complicated with CP”. Further immunohistochemical examination (**Figures 3B–F**) showed that: combined with immunohistochemical characteristics, it was consistent with a collision tumor (collision of PA and CP), mainly composed of pituitary adenoma components. The specific immunohistochemical results were: β-catenin (membranous and cytoplasmic +), CgA (partial +), CK20 (-), CK5/6 (partial +), CK7 (partial +), GFAP (partial +), Ki67 (about 1% +), P40 (partial +), P63 (partial +), Syn (+), CD56 (+), INSM1 (+), S-100 (-), TTF1 (-), PIT-1 (-), SF-1 (-), T-PIT (-). Based on the negativity of all three lineage-specific transcription factors (PIT-1, T-PIT, SF-1) and positivity for neuroendocrine markers, the adenoma component was classified as a Null-cell adenoma.

**Figure 3:**
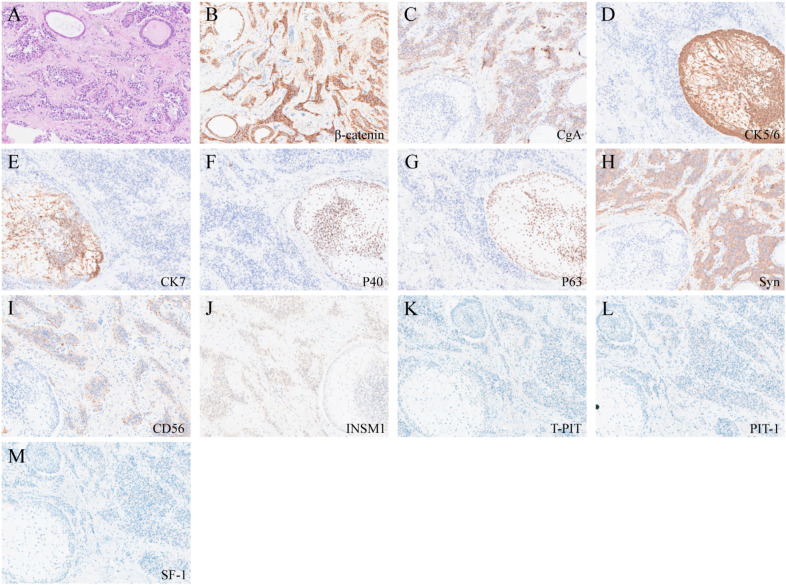
**(A)** Routine pathology; **(B)** β-catenin (membranous and cytoplasmic +); **(C)** CgA (focal +); **(D)** CK5/6 (focal +); **(E)** CK7 (focal +); **(F)** P40 (focal +); **(G)** P63 (focal +); **(H)** Syn (+); **(I)** CD56 (+); **(J)** INSM1 (+); **(K)** T-PIT (-); **(L)** PIT-1 (-); **(M)** SF-1 (-). Original magnification ×100.

The patient’s vital signs remained stable after surgery, with no obvious manifestations of hypopituitarism. Hormone reexamination on the 18th postoperative day showed the following results: serum cortisol 13.5 μg/dL, FT3 3.2 pmol/L, FT4 12.5 pmol/L, and TSH 0.38 mIU/L; other pituitary hormone levels were stable. Postoperative and post-discharge hormone replacement therapy was continuously maintained with oral hydrocortisone (20 mg daily) and levothyroxine (37.5 μg daily). The patient was discharged on the 18th postoperative day.

Follow-up evaluations were conducted after discharge. Pituitary MRI was performed 2 months after surgery and every 6 months thereafter, with no obvious evidence of tumor recurrence. The pituitary MRI obtained 2 months postoperatively (**Figures 1N–Q**) demonstrated postoperative changes of the pituitary tumor, with abnormal signal filling in the sphenoid sinus and sellar region. Contrast-enhanced imaging showed uneven enhancement in the surgical area; the pituitary stalk and pituitary gland were not clearly visualized, and patchy effusion signals were observed beneath the bilateral frontotemporal dura mater.

Hormonal follow-up 2 months after surgery showed stable endocrine function. After gradual tapering of hydrocortisone to the maintenance dose (20 mg daily), while continuing levothyroxine at 37.5 μg daily, serum cortisol was 14.2 μg/dL; FT3 was 3.3 pmol/L, FT4 was 12.8 pmol/L, and TSH was 0.41 mIU/L. Serum PRL, GH, FSH, and LH levels were all within the normal reference range for this age group.

## 3 Discussion

Preoperative pituitary MRI demonstrated imaging features consistent with a pituitary macroadenoma, including a characteristic waist sign, iso- to hypointense signal on T1-weighted imaging (T1WI), slightly hyperintense signal on T2-weighted imaging (T2WI), and homogeneous enhancement on contrast-enhanced sequences. In clinical practice, tumor consistency is often inferred from T2-weighted images by comparing the tumor signal intensity with that of a relatively stable reference region in the brainstem, such as the pons or cerebellar peduncle, with relatively hyperintense signals generally suggesting a softer tumor texture (25). Quantitative T2 signal intensity ratios have also been proposed to standardize this assessment. Based on these imaging characteristics, the lesion was preoperatively considered to have a relatively soft consistency. From an endocrine perspective, the mildly elevated preoperative prolactin level was interpreted as a pituitary stalk compression (stalk section) effect rather than a prolactin-secreting adenoma, while the presence of central hypothyroidism was consistent with hypopituitarism. Importantly, these hormonal abnormalities were not attributed to pituitary apoplexy, as no radiological evidence of hemorrhage or infarction was identified on preoperative imaging.

However, intraoperative findings revealed a markedly fibrous and tough tumor, which was inconsistent with the preoperative imaging-based assessment. Postoperative pathological examination further confirmed the presence of craniopharyngioma components intermingled with pituitary adenoma tissue. These components accounted for only a small proportion of the tumor and lacked typical radiological features such as cystic change, calcification, or heterogeneous enhancement, which likely contributed to the limitation of preoperative imaging in accurately predicting tumor consistency.

Sellar collision tumors represent a rare and poorly understood entity. To date, most knowledge regarding their clinicopathological characteristics and pathogenesis is derived from isolated case reports and small case series. The most distinguishing feature of this case, contrasting with prior reports, is the clinicoradiological and intraoperative masquerade of the collision tumor.

Typically, collision tumors present with a ‘snowman’ shape or distinct cystic/solid components on MRI. However, our case presented as a radiologically homogeneous lesion with a ‘waist sign’, mimicking a simple macroadenoma. Furthermore, the fibrous and tough texture encountered intraoperatively likely obscured the interface between the two pathologies. This ‘intermingled growth’ pattern created a diagnostic blind spot, where the dual pathology was an unanticipated histological discovery. This underscores the necessity of routine comprehensive immunohistochemical screening for all sellar masses, even those appearing morphologically uniform.

The surgical management of this case presented four distinct challenges necessitating specific technical strategies. First, the tumor’s unexpectedly fibrous consistency precluded standard suction decompression, requiring sharp piecemeal resection; this textural uniformity also obscured the macroscopic interface between the collision components, contributing to the diagnostic complexity. Second, the significant vertical suprasellar extension (Hardy Grade B, Knosp Grade 2) necessitated an Extended Endoscopic Transsphenoidal Approach (EETSA) with incision of the anterior reflection of the diaphragma sellae to mobilize the pituitary gland and expose the tumor dome. Third, despite dense adhesions and significant cavernous sinus hemorrhage, meticulous dissection along the arachnoid plane ensured the functional preservation of the pituitary stalk, optic chiasm, and superior hypophyseal arteries. ly, to address the large skull base defect, a rigorous multilayered reconstruction protocol utilizing a vascularized nasoseptal flap and postoperative lumbar drainage was implemented, successfully preventing cerebrospinal fluid leakage.

Literature reports indicate that the incidence of collision tumors between PA and RCC is only 0.9%. Their MRI mostly presents as cystic-solid lesions, which are difficult to differentiate from other sellar lesions such as craniopharyngioma (26). The collision between PA and CP is even rarer, posing multiple challenges to clinical diagnosis. Meanwhile, it causes the tumor to have a tough texture during surgery, significantly increasing the difficulty of resection. It can be seen that conventional preoperative neuroimaging examinations such as MRI cannot accurately determine the tumor density of sellar collision tumors, making it difficult to select an appropriate surgical approach. Moreover, such tumors mostly rely on postoperative pathology for confirmation before surgery, which may easily delay treatment planning (27). Therefore, comprehensive preoperative evaluation of tumor density is extremely important. Studies have pointed out that MR elastography can analyze the density of pituitary adenomas preoperatively (27), which helps improve surgical precision.

Although both CP and PA are relatively common tumors in the parasellar region, the simultaneous presence (i.e., coexistence) of these two pathological types in the same anatomical site is extremely rare. A comprehensive literature search was conducted in PubMed up to October 2025 using the following keywords: (“pituitary adenoma” OR “PA”) AND (“craniopharyngioma” OR “CP”) AND (“collision tumor”). Only peer-reviewed English-language case reports describing histologically confirmed sellar collision tumors were included. Articles describing composite tumors, RCC-associated lesions, or insufficient pathological confirmation were excluded. Two authors independently screened titles and abstracts, and discrepancies were resolved by consensus. A total of 18 cases met the inclusion criteria and were summarized in **Table 1**.

**Table 1:** Summary of clinical characteristics, pathological subtypes in patients with PA and CP.

Author & year	Age (yrs)/Gender	Pathological subtypes PA	Pathological subtypes CP
Finzi, G. et al., 2014 (13)	75/F	Silent subtype 2 ACTH	Adamantinomatous
Gokden, M. et al., 2009 (14)	47/M	Non-functional	Adamantinomatous
Jin, G. et al., 2013 (15)	47/F	Non-functional	Adamantinomatous
Karavitaki, N. et al., 2008 (16)	50/M	Gonadotroph PA	Adamantinomatous
Moshkin, O. et al., 2009 (17)	12/M	Silent subtype 3	Adamantinomatous
Kurnukhina et al., 2025 (27)	61/M	Non-functional	Papillary
Gong, L. et al., 2022 (36)	54/F	Non-functional	Adamantinomatous
Miyazaki, T. et al., 2019 (28)	48/M	Non-functional	Adamantinomatous
Kikuta, H. et al., 2023 (31)	54/M	Dual-hormone (PIT-1+TSH/SF-1+FSH)	Adamantinomatous
Shareef, Z. et al., 2020 (30)	60/M	Recurrent non-functional	Adamantinomatous
Snyder, R. et al., 2019 (33)	49/F	ACTH-positive	Adamantinomatous
Bteich, F. et al., 2020 (35)	35/M	Non-functional	Papillary (1st report, BRAF V600E+)
Hasegawa, H. et al., 2021 (29)	51/M	Silent gonadotroph (FSH+)	Adamantinomatous
Shakally, A. et al., 2020 (39)	41/M	Non-functional PA (FSH+)	Adamantinomatous
Mancini, A.J. et al., 2024 (32)	49/M	GH-secreting (PIT-1+)	Papillary
Burgos-Sosa, E. et al., 2024 (37)	57/M	Non-functional	Papillary (BRAF V600E+)
Zhao, Y. et al.,2025 (38)	75/M	Non-functional PA (focal LH/FSH+, Ki-67 = 2%)	Adamantinomatous
McLean, et al., 2025 (34)	49/F	Gonadotroph PA (FSH/LH+/SF-1+)	Adamantinomatous(β-catenin+)

ACTH, Adrenocorticotropic Hormone; CP, Craniopharyngioma; FSH, Follicle-Stimulating Hormone; GH, Growth Hormone; LH, Luteinizing Hormone; PA, Pituitary Adenoma; PIT-1, Pituitary Transcription Factor 1; SF-1, Steroidogenic Factor 1; TSH, Thyroid-Stimulating Hormone.

A review of these case reports revealed that most of the PA documented in the literature were non-functional (10 cases). Functional PAs included gonadotropin-secreting type (FSH/LH+, 4 cases), adrenocorticotropic hormone-secreting type (ACTH+, 2 cases), growth hormone-secreting type (GH+, 1 case), dual-hormone type (TSH/FSH+, 1 case), and some PAs were silent subtypes. The vast majority of CP were of the adamantinomatous type (14 cases), while the papillary type was less common (only 4 cases), among which 2 cases were detected with BRAF V600E gene mutation. Combined with preoperative laboratory examinations and postoperative pathological immunohistochemical findings, this case showed that the PA was non-functional and the CP was of the adamantinomatous type, which is consistent with the results of the literature review.

The literature also found that in cases of collision between PA and CP, the two tumor components in some cases were integrated without a relatively clear boundary, which was also observed in the pathological results of this case. In this regard, the academic community has proposed several hypotheses:

### 3.1 Hypothesis of multidirectional differentiation from a common progenitor cell

This hypothesis suggests that the multiple tumor components of sellar collision tumors originate from the same embryonic residual tissue (e.g., Rathke’s pouch). Abnormal cell differentiation during embryonic development leads to the simultaneous occurrence of multiple types of tumors. The epithelial lining of RCC undergoes a transformation process from ciliated columnar epithelium to squamous epithelium, with a structure similar to tracheobronchial epithelium and progenitor cell characteristics. Under the influence of external factors such as hormonal environment and inflammatory stimulation, these progenitor cells may undergo abnormal differentiation, thereby forming mixed tumors such as RCC with PA and RCC with CP. Finzi, G. et al. (13) observed “hybrid cells” through electron microscopy; Karavitaki, N. et al. (16) reported that the two components share a common ancestral cell in terms of embryonic origin; and Gokden, M. et al. (14) observed histological regions where adenoma cells transition to craniopharyngioma cells. All these provide evidence for this hypothesis.

### 3.2 Hypothesis of induction by local microenvironment

This hypothesis holds that an existing tumor in the sellar region can induce secondary tumor transformation in surrounding tissues by altering the local microenvironment (e.g., hormone levels, inflammatory response). Articles such as Jin, G. et al. (15) and Miyazaki, T. et al. (28) mention that if a CP compresses the pituitary stalk, it may block the inhibitory effect of hypothalamic dopaminergic signals on the pituitary gland, leading to abnormal proliferation of pituitary prolactin cells and thus the formation of a prolactinoma. Hasegawa, H. et al. (29) reports that the pituitary adenoma compresses the pituitary stalk and the hormonal paracrine effect promotes the proliferation of craniopharyngioma. Shareef, Z. et al. (30) proposes that chronic inflammation around RCC may cause metaplasia of the cyst wall epithelium, and further mutations may form RCC and PA; Kikuta, H. et al. (31) shows that changes in hormone levels after surgery may stimulate the growth of specific PA. In addition, Mancini, A. J. et al. (32) and Snyder, R. et al. (33) all report that abnormal hormones such as growth hormone and prolactin may promote the formation of collision tumors.

### 3.3 Hypothesis of abnormal differentiation of multipotent stem cells

This hypothesis suggests that sellar collision tumors originate from multipotent stem cells with multidirectional differentiation potential. These stem cells may differentiate into different types of tumor cells simultaneously due to gene mutations or regulatory abnormalities. McLean, L. et al. (34) and Bteich, F. et al. (35) mentions that if multipotent stem cells in the pituitary undergo gene mutations such as BRAF-V600E and β-catenin, they may differentiate into PA cells and CP cells simultaneously, thereby forming a collision tumor. Moreover, the pathological examination of this case showed no clear boundary between the two tumor components, supporting the view of a common stem cell origin. Gong, L. et al. (36) points out that stem cells in the embryonic residual tissue of the sellar region may differentiate into malignant AT/RT cells and craniopharyngioma cells simultaneously due to SMARCB1 gene deletion or β-catenin mutation; Burgos-Sosa, E. et al. (37) and Kurnukhina, M. Y. et al. (27) believe that it is related to the embryonic residual tissue of Rathke’s pouch. Different cell populations in the residual tissue undergo neoplastic transformation respectively to form collision tumors, and relevant cases all support this hypothesis.

In addition, some collision tumor cases show clear boundaries in pathology and imaging. Zhao, Y. et al. (38) suggests that the different tumor components of such sellar collision tumors have completely independent origins. Due to the narrow anatomical space of the sellar region, tumors in multiple locations grow randomly and simultaneously, occupying the same area, with no direct pathophysiological connection between them. Molecular detection results also show that the pathogenic pathways of PA and CP are completely independent: among craniopharyngiomas, the adamantinomatous type (ACP) is associated with β-catenin pathway activation (exon 3 point mutation), and the papillary type (PCP) is characterized by BRAF-V600E mutation; while the pathogenesis of pituitary adenoma is related to abnormal cell cycle pathways (Rb/p16/Cyclin D1/CDK4), abnormal GADD45γ regulation, and MEG3 gene mutation. There is no common driver gene mutation between the two, which rules out the possibility of a single pathway inducing the occurrence of multiple tumors and further supports the view that the tumors occur independently. Shakally, A. et al. (39) reports that the FSH-positive non-functioning pituitary adenoma (NFPA) and the adamantinomatous craniopharyngioma both originate from the Rathke’s pouch but undergo independent clonal proliferation. The craniopharyngioma relies on the activation of the WNT/β-catenin signaling pathway (characterized by nuclear β-CATENIN co-localization with LEF1), while this pathway is not activated in the adenoma. The recurrence of the adenoma is due to the reactivation of proliferation of dormant residual cells after the first surgery.

## 4 Conclusion

This article presents a rare sellar collision tumor composed of pituitary adenoma and craniopharyngioma and integrates a focused review of the pathogenetic mechanisms based on previously reported cases. Preoperative MRI only demonstrated features of a pituitary macroadenoma. The craniopharyngioma component, which lacked typical imaging characteristics, was not detected due to its small size and integration with the adenoma and was confirmed only by postoperative pathology. The tumor was resected via endoscopic transsphenoidal surgery. Postoperatively, the patient received hormone replacement therapy and was discharged on day 18. Long-term follow-up showed no recurrence. This case aligns with literature findings that most such collision tumors involve non-functional adenomas and adamantinomatous craniopharyngiomas, and it contributes to optimizing the diagnosis and treatment of this rare disease.

## Data Availability

The original contributions presented in the study are included in the article/supplementary material. Further inquiries can be directed to the corresponding authors.
